# 

**Published:** 1911-12

**Authors:** 


					﻿PUBLISHED MONTHLY.
SUBSCRIPTION $1.00
ATLANTA
JOURNAL-RECORD
OF \1II)KJNE; =
(Atlanta Medical and Surgical Journal, Established
and Southern Medical Record, Established 1870.	\ ^*>41—Hcflr
Vol. LVII.	Atlanta, Ga., December 1911.	No. 9
				

